# Real-Time Monitoring of Photocytotoxicity in Nanoparticles-Based Photodynamic Therapy: A Model-Based Approach

**DOI:** 10.1371/journal.pone.0048617

**Published:** 2012-11-07

**Authors:** Hamanou Benachour, Thierry Bastogne, Magali Toussaint, Yosra Chemli, Aymeric Sève, Céline Frochot, François Lux, Olivier Tillement, Régis Vanderesse, Muriel Barberi-Heyob

**Affiliations:** 1 Université de Lorraine, Centre de Recherche en Automatique de Nancy (CRAN), UMR 7039, Vandœuvre-lès-Nancy, France; 2 CNRS, Centre de Recherche en Automatique de Nancy (CRAN), UMR 7039, Vandœuvre-lès-Nancy, France; 3 Inria, Biologie, Génétique et Statistiques (BIGS), UMR 7502, Institut Elie Cartan Nancy (IECN), Vandœuvre-lès-Nancy, France; 4 CNRS, Laboratoire des Réactions et Génie des Procédés (LRGP), UPR 3349, Nancy, France; 5 CNRS, GdR 3049 “Médicaments Photoactivables - Photochimiothérapie (PHOTOMED)”, France; 6 Université Claude Bernard Lyon 1, Laboratoire de Physico-Chimie des Matériaux Luminescents (LPCML), UMR 5620, Villeurbanne, Lyon, France; 7 CNRS, Laboratoire de Physico-Chimie des Matériaux Luminescents (LPCML), UMR 5620, Villeurbanne, Lyon, France; 8 Université de Lorraine, Laboratoire de Chimie-Physique Macromoléculaire (LCPM), UMR 7568, Nancy, France; 9 CNRS, Laboratoire de Chimie-Physique Macromoléculaire (LCPM), UMR 7568, Nancy, France; 10 Centre Alexis Vautrin, Centre Régional de Lutte Contre le Cancer (CRLCC), Vandœuvre-lès-Nancy, France; Florida International University, United States of America

## Abstract

Nanoparticles are widely suggested as targeted drug-delivery systems. In photodynamic therapy (PDT), the use of multifunctional nanoparticles as photoactivatable drug carriers is a promising approach for improving treatment efficiency and selectivity. However, the conventional cytotoxicity assays are not well adapted to characterize nanoparticles cytotoxic effects and to discriminate early and late cell responses. In this work, we evaluated a real-time label-free cell analysis system as a tool to investigate *in vitro* cyto- and photocyto-toxicity of nanoparticles-based photosensitizers compared with classical metabolic assays. To do so, we introduced a dynamic approach based on real-time cell impedance monitoring and a mathematical model-based analysis to characterize the measured dynamic cell response. Analysis of real-time cell responses requires indeed new modeling approaches able to describe suited use of dynamic models. In a first step, a multivariate analysis of variance associated with a canonical analysis of the obtained normalized cell index (NCI) values allowed us to identify different relevant time periods following nanoparticles exposure. After light irradiation, we evidenced discriminant profiles of cell index (CI) kinetics in a concentration- and light dose-dependent manner. In a second step, we proposed a full factorial design of experiments associated with a mixed effect kinetic model of the CI time responses. The estimated model parameters led to a new characterization of the dynamic cell responses such as the magnitude and the time constant of the transient phase in response to the photo-induced dynamic effects. These parameters allowed us to characterize totally the *in vitro* photodynamic response according to nanoparticle-grafted photosensitizer concentration and light dose. They also let us estimate the strength of the synergic photodynamic effect. This dynamic approach based on statistical modeling furnishes new insights for *in vitro* characterization of nanoparticles-mediated effects on cell proliferation with or without light irradiation.

## Introduction

Drug delivery systems able to selectively target diseased tissues with minimum side effects remain a major challenge in the development of efficient pharmacological treatments for cancer. Currently, one exciting approach towards the development of suitable delivery systems, that can circumvent the physiological barriers and mechanisms that may compromise efficiency of the treatment, involves the use of nanocarrier systems tailored to selectively deliver active molecules to target tissues [1]–[3]. Numerous nanosized objects have been explored in many biomedical applications because of their novel properties, such as their high surface to volume ratio, their surface tailorability and their multifunctionality [4], [5]. As drug carriers, nanoparticle systems consist of different composition types and molecular structures within the active molecules can be entrapped or conjugated covalently. Accumulating investigations have demonstrated the potential of nanoparticles in clinical applications, and especially in the development of anticancer therapies [1], [6], [7]. Recent researches in imaging and diagnostic applications of nanoparticles have also clearly illustrated the direct impact of such nanomaterials on the possibility to investigate the biodistribution and pharmacokinetic of nanoparticles-based drug carrier systems, both *in vitro* and *in vivo.* Some of the recent researches using nanoparticles as magnetic resonance imaging (MRI) contrast agents, fluorescence imaging agents, and potential carriers for drug delivery, have been published [8]–[12]. In this field, our group described multifunctional fluorescent nanoparticles containing a gadolinium oxide core as very attractive system, aiming at combining both imaging (fluorescence, MRI) and therapy (X-ray therapy, photodynamic therapy (PDT)) techniques [13], [14].

PDT for cancer involves the uptake of a photoactivatable drug, also known as photosensitizer or photosensitizing agent, by cancer tissue followed by localized photoirradiation with visible light at appropriate wavelength and dose. Photoactivation of the photosensitizer results in the formation of photosensitizer excited state that transfers its energy to surrounding molecular oxygen and leads to the local production of reactive oxygen species (ROS), such as singlet oxygen (^1^O_2_). Such highly cytotoxic species induce cellular damage leading to an alteration of the tumor vasculature and tumor cells death [15]–[17]. PDT is usually described as an alternative treatment modality for cancer and various other diseases [17], [18]. Increasing evidences indicate that the use of nanoparticles as carriers of photoactivatable molecules seem to be a very promising approach to satisfy a large number of the requirements for an ideal targeted PDT [8], [19], [20], [14].

The commonly used cell viability assays to evaluate *in vitro* cytotoxicity and photosensitizing ability of the photoactive compounds are based on the determination of cell metabolism activity. These include several methods mainly based on the quantification of intracellular adenosine triphosphate, or the reduction of tetrazolium salts to formazan dyes by mitochondrial dehydrogenases in viable cells. However, these methods, known as single end-point qualitative measures, are incompatible with experimental treatments that may directly influence and/or modulate cellular metabolism, mitochondrial activity, mitochondria intracellular mass [21], [22], or cell adhesion, proliferation and migration abilities. It has been reported that some substances, predominantly redox active compounds, can react with formazan dyes, bringing misleading information on cell viability [23]. These limitations are furthermore relevant, in the case of nanoparticles systems that present a real lack of information regarding their potential toxicity [24]. It is known that nanoparticles as well as nanoparticles-generated ROS can interact with the assays reagents and interfere with the readout [25], [26]. Moreover, growing reports suggest the importance of exposure/interaction time factor to take into account in studying nanoparticles-mediated cytotoxicity (for review see, [25]). Such investigations highlight the need to monitor a time-dependent cell response. Furthermore, investigation of early and late effects of anti-tumor PDT generally involves the combination of various single end-point cell viability assays, which may generate discrepancies in data [27], [28]. Winther et *al.*, [28] reported inconsistency in cell survival assessed by trypan blue exclusion and the number of clonogenic cells, following Photofrin II-PDT on a retinoblastoma-like cell line.

In the present study, we have analyzed for the first time, dynamic data obtained from a real-time cell analysis system in order to investigate the cyto- and photocyto-toxic effects of multifunctional nanoparticles on human breast cancer cells. This system based on impedance measurement allows on-line and continuous monitoring of cellular events. Moreover, as label-free method, the impedance-based cell analysis could be a more valuable approach since cell metabolism based assay reagents may interfere with treatment, particularly nanoparticles [25], [26]. It measures electrical impedance across interdigitated micro-electrodes integrated on the bottom of cell culture plates. Impedance data are automatically converted to Cell Index (CI) values that are defined as relative change in electrical impedance created by attached cells (cellular status), and are directly proportional to cell number, cell proliferation and growth, cellular adhesion, cell size and morphology, as well as intercellular interactions. As cells detach and die (*i.e.* during cytotoxic events) the cell-covered area reduces and CI values decrease [29], [30]. Thus, the impedance-based cell analysis offers a multiparametric biological analysis of cell activities in real-time, providing extensive rich dynamic data about the global cell proliferation and growth dynamics.

Photodynamic activity using nanoparticles can result in the alteration of cell activities and the loss of cell viability related to photosensitizer concentration and light dose. The dynamic analysis of the nanoparticles-induced photocytotoxicity showed discriminant profiles of cell response kinetics in a photosensitizer concentration- and light dose-dependent manner. Since the real-time analysis generates a large amount of time profiles and rich dynamic information, the challenge was then to provide statistical techniques able to reduce the dimension of the inference problem and to extract the meaningful information characterizing the photodynamic effects. The main modeling challenges were firstly, to associate statistical design of experiments and biological kinetics modeling and secondly, to find out a model structure able to fit all the different dynamic cell responses, overtaking *plethora* of profiles. By using a parametric modeling analysis of the obtained photocytotoxicity time profiles, we deduced dynamic model parameters that completely characterized the dynamic cell response. These model parameters described distinct phases of the photodynamic response, and provided numeric information about the dynamic behaviour of cell response. Moreover, the real-time impedance based analysis of cell response allowed discrimination between early and late cellular effects thanks to the continuous monitoring. A strong relationship was also shown between the different dynamic parameters.

## Results

### Nanoparticles-induced Dark Cytotoxicity *in vitro*

**Figure 1:**
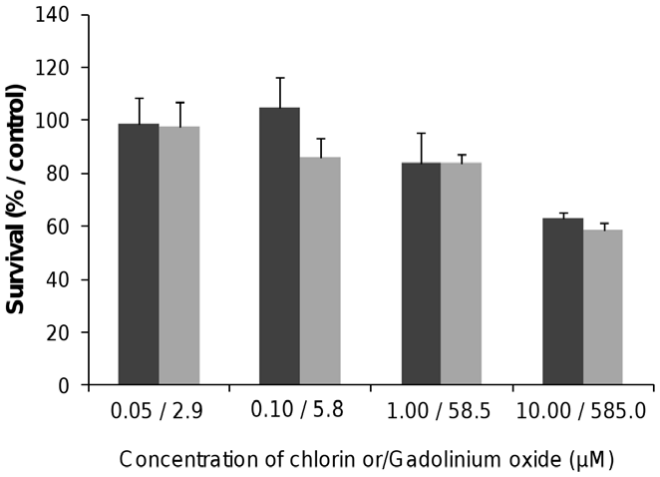
Dark cytotoxicity without light exposure of nanoparticles-grafted photosensitizer (NP-PS) or control nanoparticles (NP) using MTT assay. MDA-MB-231 cells were exposed to NP-PS (dark grey) or NP (clear grey) at the mentioned concentrations for 24 h. Cell viability was evaluated by MTT assay (data points show the mean ± S.D., n = 6).

Metabolic activity. Following exposure to the multifunctional peptide-targeted ultrasmall silica-based nanoparticles, cell viability was first determined using the colorimetric MTT metabolic activity assay on human breast cancer cells. We optimized H-Ala-Thr-Trp-Leu-Pro-Pro-Arg-OH (ATWLPPR) peptide-targeted silica-based nanoparticles to target the tumor vasculature through the vascular receptor neuropilin-1 (NRP-1) [14]. These hybrid non-biodegradable nanoparticles consisted of a gadolinium chelates as MRI contrast agent, a silica shell containing the covalently grafted chlorine-type photosensitizer molecules, DOTAGA (1,4,7,10-tetraazacyclododecane-1,4,7,10-tetraacetic acid) as an active chelator surfactant and a surface-localized ATWLPPR peptide as targeting units. Dark cytotoxicity (without light irradiation) was first assessed by MTT test in response to increasing concentrations of nanoparticles-grafted photosensitizer molecules (NP-PS) or photosensitizer-free nanoparticles (NP) (from 0.05 to 10.00 µM of chlorin, corresponding to 2.9 to 585.0 µM of gadolinium, respectively). As shown on **Fig. 1**, 48 h after nanoparticles exposure, cytotoxic effect was evidenced only with the higher concentration (10.00 µM/585.0 µM), leading to a reduction of 37% and 41% of cell survival for NP-PS and NP, respectively. No cytotoxic effect was measured (mean cell viability superior to 80%) for the cells exposed with concentrations ranging from 0.05 to 1.00 µM for both nanoparticle groups (NP-PS and NP) compared to untreated cells (**Fig. 1**).

#### Real-time impedance-based analysis

**Figure 2:**
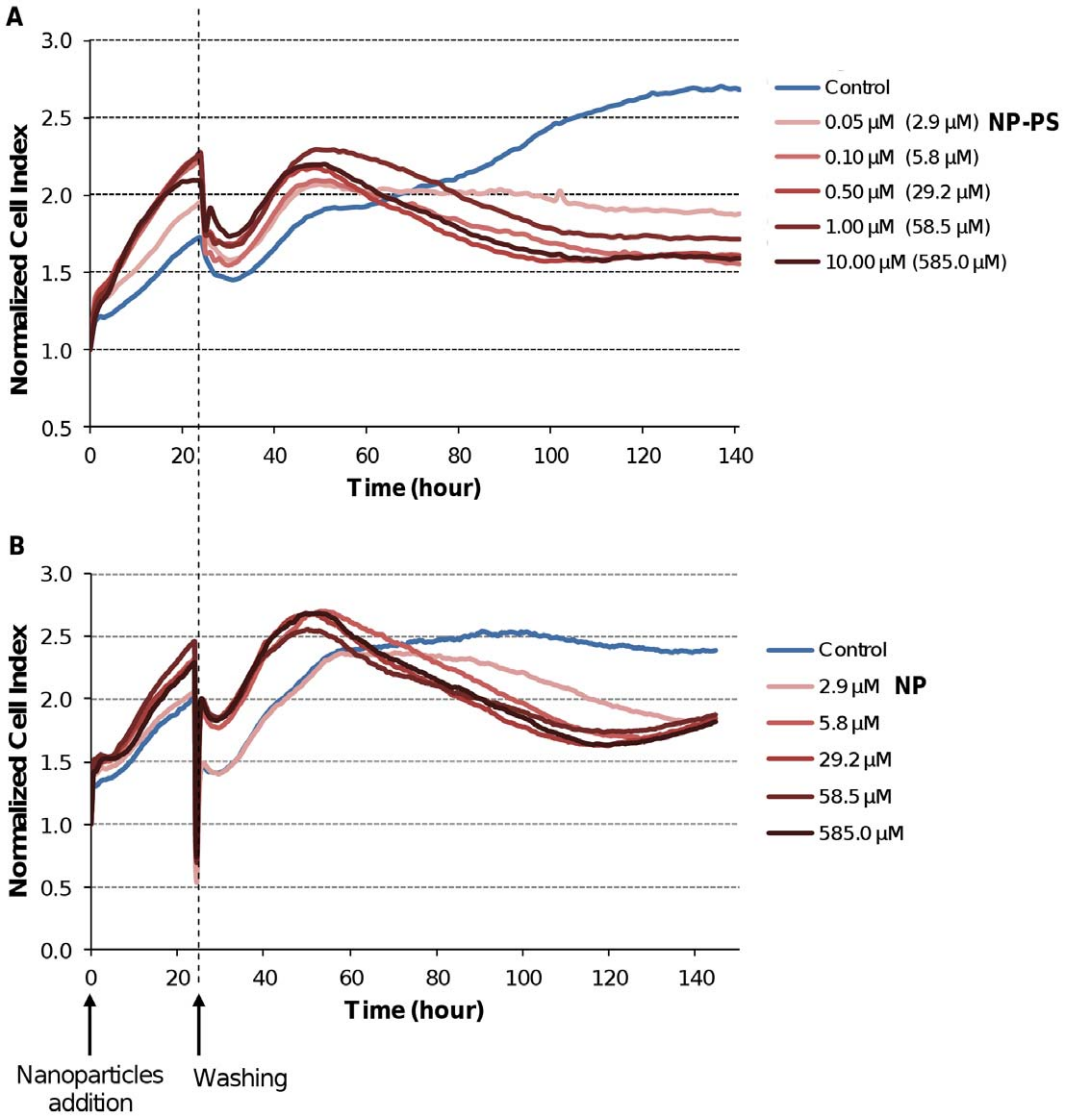
Normalized cell index (NCI) kinetics of the MDA-MB-231 cells exposed to nanoparticles without light irradiation. The cells were exposed to the indicated concentrations of (A) nanoparticles-grafted photosensitizers (NP-PS), or (B) photosensitizer-free nanoparticles (NP) for 24 h before washing. Cell Index (CI) was monitored during 143 h after nanoparticles exposure. Reported data are the means of six replicates.

Real-time cell analysis (RTCA) system measures impedance-based signals and provides dynamic information. The cellular response was continually monitored for 143 h in darkness from the time of nanoparticles were added. As shown in **Fig. 2**, electrical impedance measurements from adherent cells, assessed by NCI kinetics, showed no decrease in CI values whatever the nanoparticles (NP and NP-PS) concentration used as compared to untreated cells. A transient decrease of CI was recorded at 24 h-post addition, related to the temporary interruption of impedance measurement during the washing step performed to washing off the un-internalized nanoparticles (**Fig. 2**). However, ∼60 h after nanoparticles exposure, CI kinetics were clearly modified, showing an evident time-dependent decrease of CI for all the concentrations tested as compared to control (**Fig. 2**).

#### Statistical modeling of the dynamic cell response

**Figure 3:**
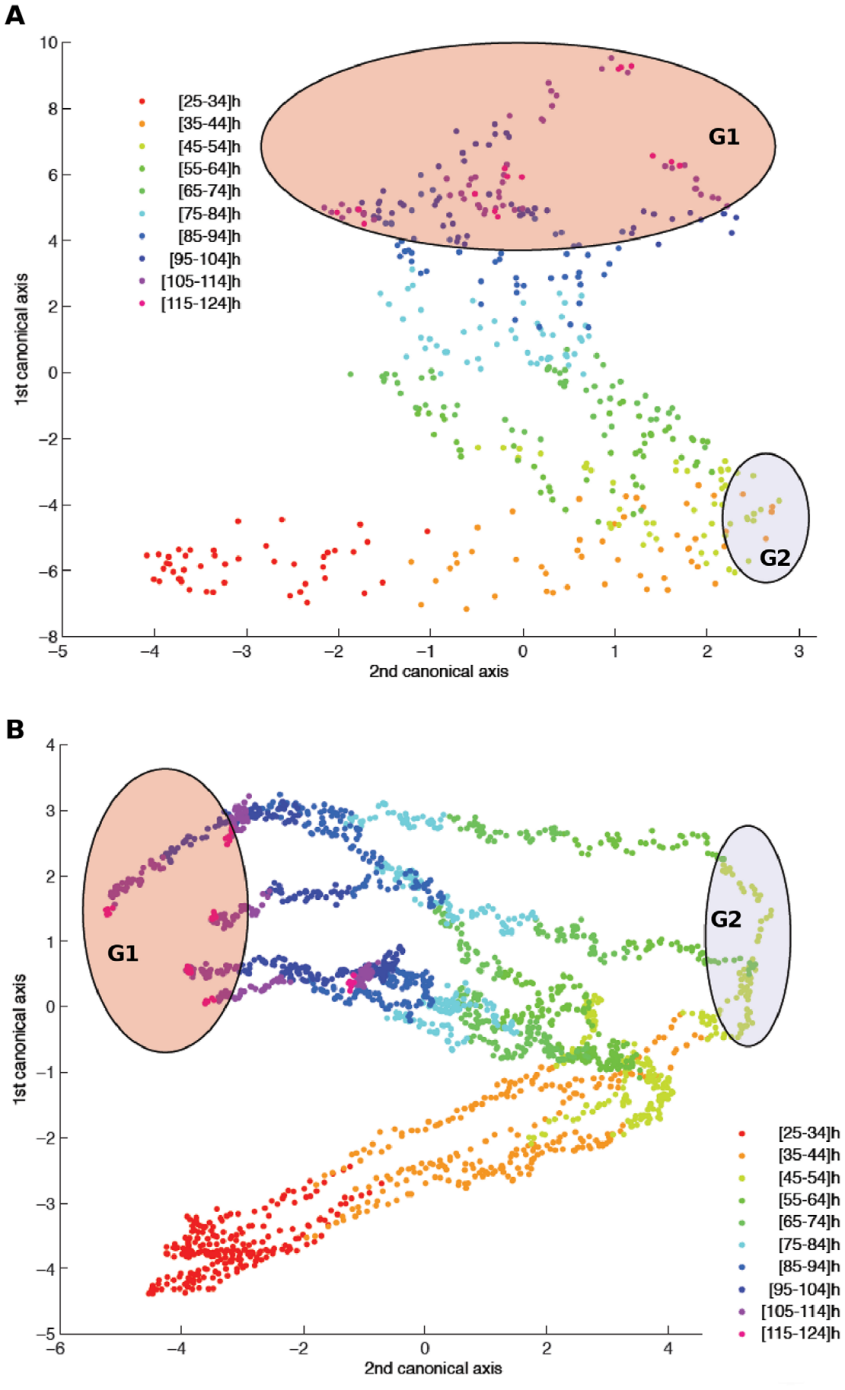
Canonical representation of a multivariate ANOVA with respect to the measurement time instants. The two canonical axes contain more than 95% of the total information contained in the time profiles over the study range [25–120] h presented in Figures 4A**–**B. According to this synthetic representation, there are two significant time regions of interest, described by blues and red ellipses. These two regions correspond to the most distant time points (with respect to the initial time instant t_0_) for the two axes. These two time regions of interest are reported in the time plots of Figures 4A**–**B with the same color code. Data used in this statistical analysis have been previously normalized by fixing the cell index (CI) values at 1 at initial time t = 0 for all the cell cultures.

**Figure 4:**
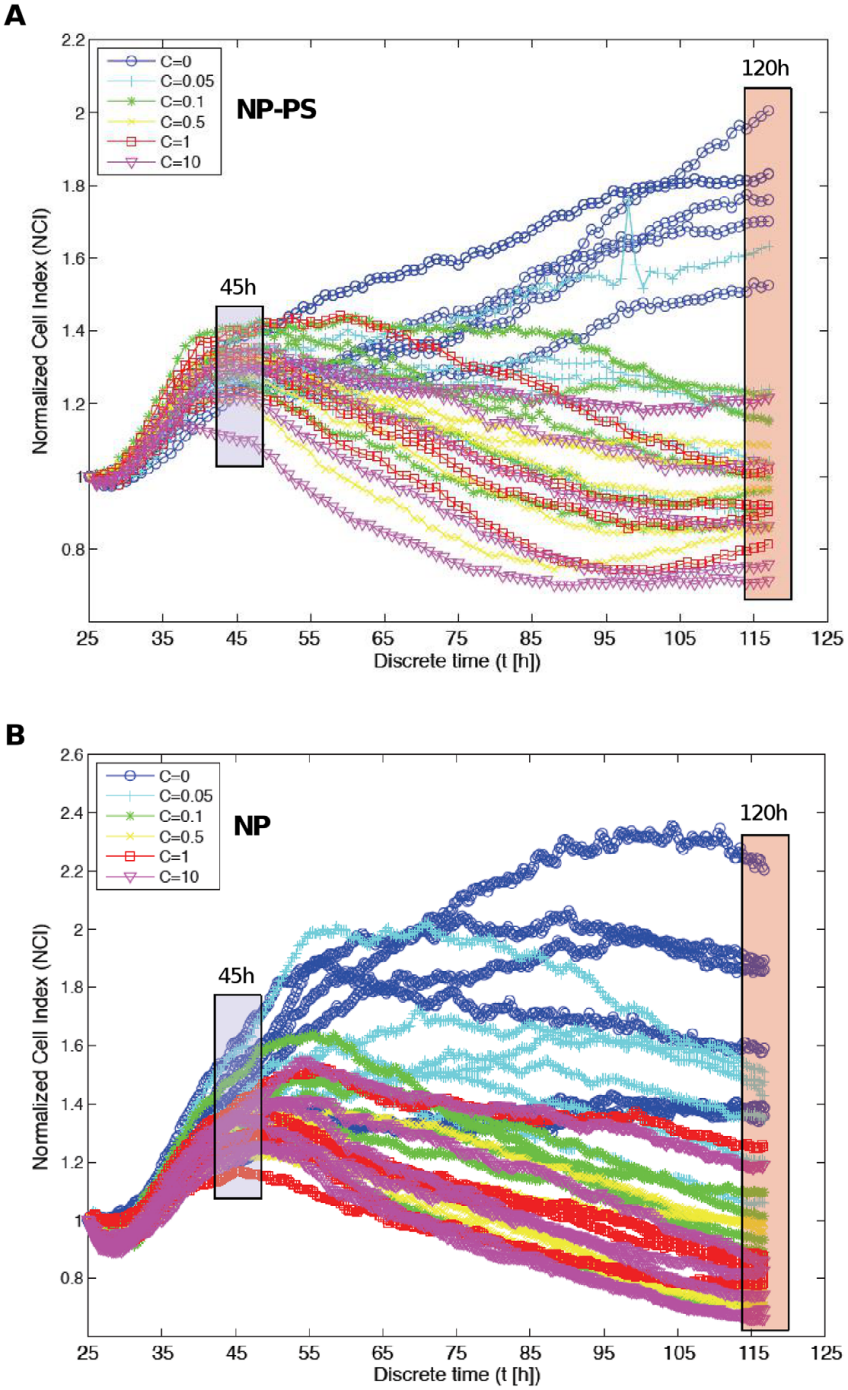
Study area [25–120] h of the normalized cell index (NCI) kinetics for different concentrations of nanoparticles. MDA-MB-231 cells were exposed to various concentrations of A) nanoparticles-grafted photosensitizer (NP-PS) or B) photosensitizer-free nanoparticles (NP) as described in Figure 2. Two time regions of interest, colored in blue and red, were identified by a statistical analysis (results in Figures 3A**–**B) as the most informative ones: around 45 h and 120 h after nanoparticles exposure. At t_0_ = 25 h (beginning of the study area) the NCI values are normalized at one.

**Figure 5:**
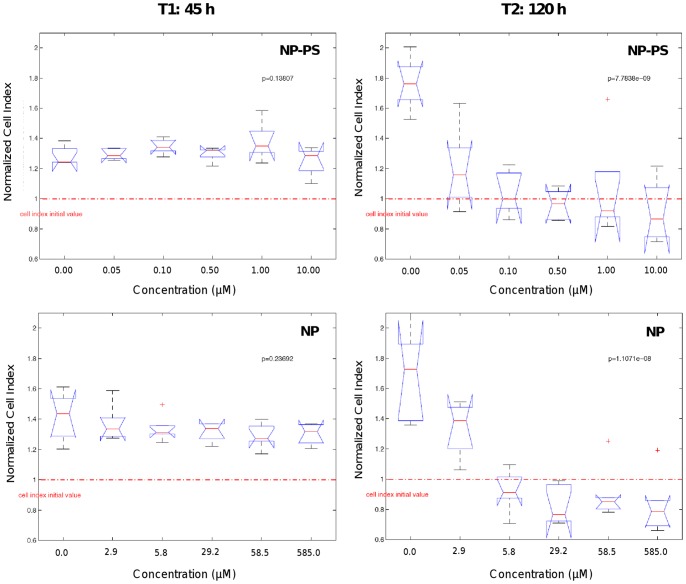
Analysis of variance of the normalized cell index (NCI) values at times T1 = 45 h and T2 = 120 h. This analysis was performed with respect to the indicated six concentration groups of photosensitizer into NP-PS or the six concentration groups of nanoparticles without photosensitizer (NP) at times T1 = 45 h (left panel) and T2 = 120 h (right panel).

A multivariate analysis of variance (MANOVA) associated with a canonical analysis was applied to the NCI values in order to identify the time regions of interest, for which the largest difference of NCI between the six groups of concentration may be observed, *i.e.* the most informative parts (**Fig. 3** **)**. All measurement time instants (from 25 to 120 h following nanoparticles exposure) were projected into a canonical map, which summarized more than 95% of the total variance information contained in the original data. The analysis of the first canonical axis revealed the importance of a first time region of interest (group 1; G1) corresponding to the end part of the experimentation period (T ≈ 120 h). According to the second axis, another group (G2) was also selected around T ≈ 45 h. These two time regions were identified for the two sets of experimentation (**Fig. 3A–B**). Two one-way ANOVA were then performed upon the NCI values at the identified time instants T1 = 45 h and T2 = 120 h for the six groups of concentration in order to assess nanoparticles concentration-dependent cellular effects (**Fig. 4** **)**. At T1 = 45 h, no statistically significant mean variation was detected between the six concentrations of NP-PS and NP. However, at T2 = 120 h, a statistically significant decrease of NCI values was observed in a concentration-dependent manner **(** **Fig. 5** **)**. Most of the NCI median values were below the NCI initial level, revealing a significant cytotoxic effect (p = 7.78e-09 and 1.11e-08) of the NP-PS and NP, respectively. Such an alteration of cell proliferation was measured for both nanoparticle sets, indicating no direct effect of the photosensitizer molecules conjugated in nanoparticles **(** **Fig. 5** **)**.

### Photocytotoxicity Profiles

**Figure 6:**
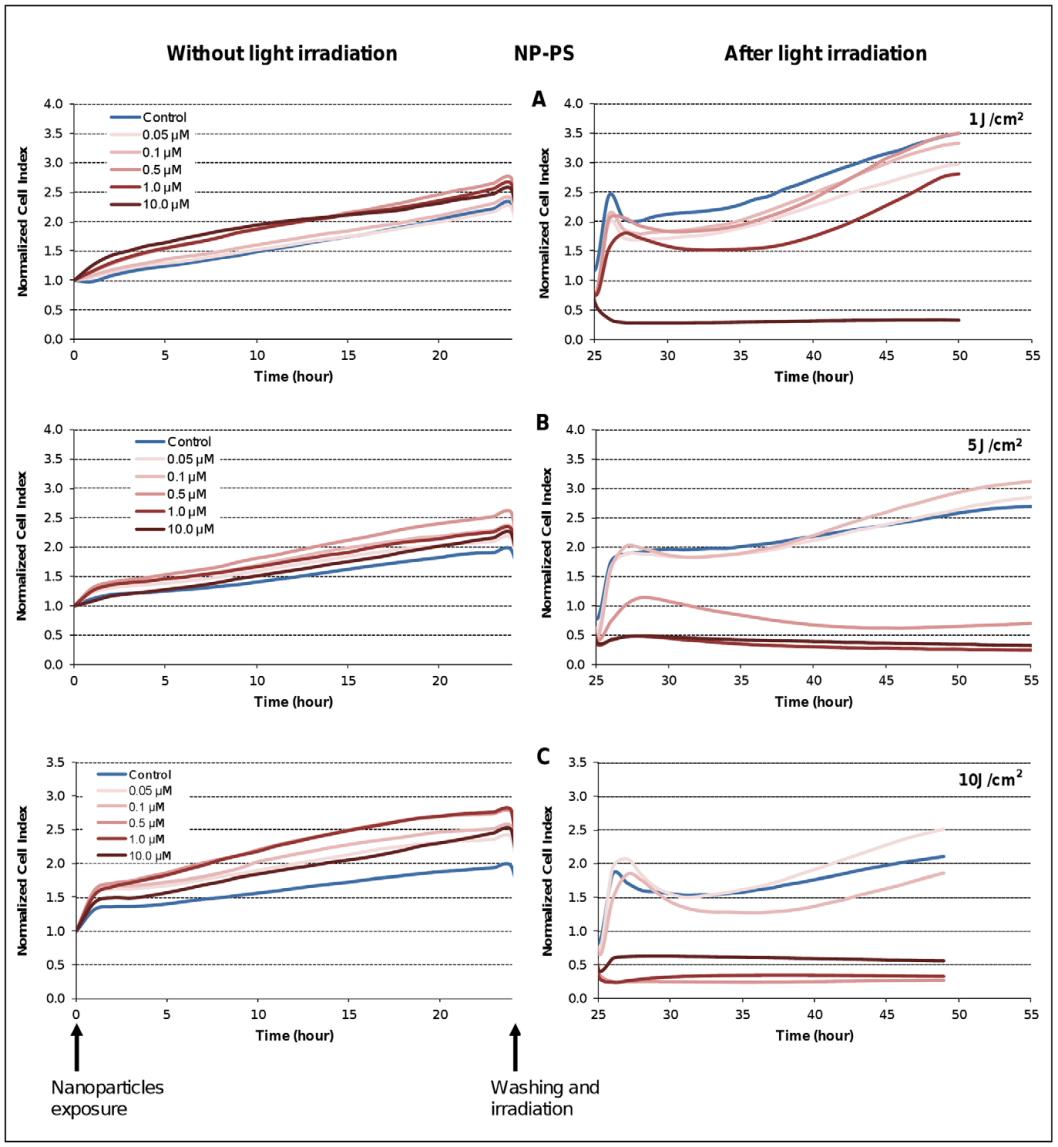
Kinetics of photo-induced cytotoxicity of nanoparticles-grafted photosensitizer (NP-PS) according to real-time impedance analysis. MDA-MB-231 cells were monitored for 24 h during interaction with NP-PS at the indicated concentrations of photosensitizer (left panel) before washing and light irradiation at 1 J/cm^2^ (A), 5 J/cm^2^ (B) or 10 J/cm^2^ (C) (right panel). Presented cell index (CI) values are the mean of 6 replicates.

**Figure 7:**
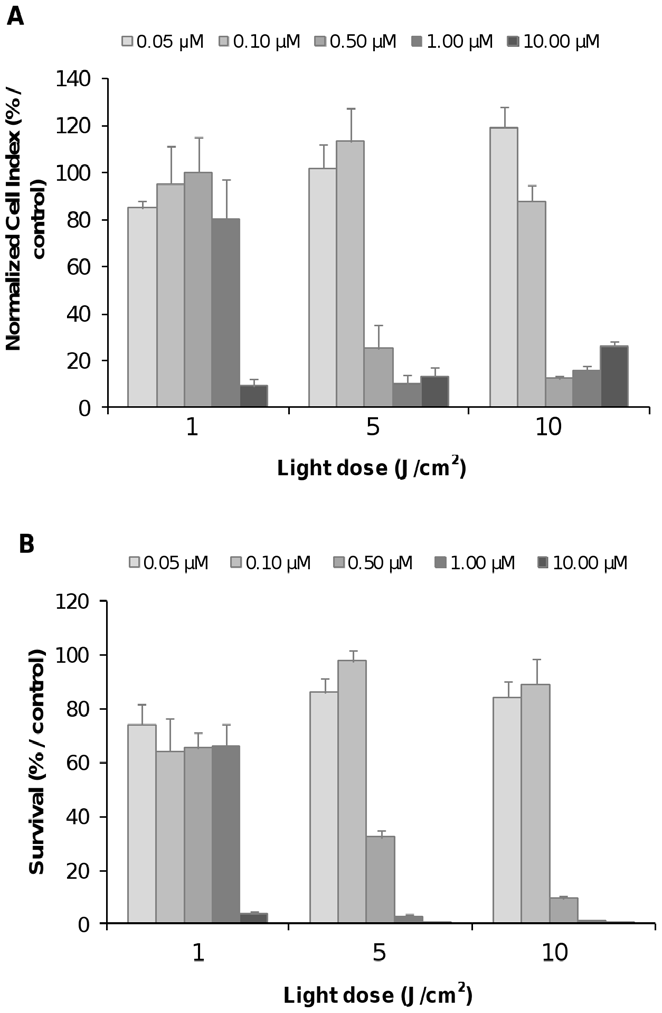
Photo-induced cytotoxicity of nanoparticles-grafted photosensitizer using impedance analysis and WST-1 test at 24 h post-irradiation. For impedance-based analysis (A), the MDA-MB-231 cells were exposed to various concentrations of nanoparticles-grafted photosensitizer (NP-PS) (from 0.05 to 10 µM) for 24 h followed by a washing step before exposition to the indicated doses of light. At 24 h post-irradiation, WST-1 test (B) was carried out on the same E-Plate. Data are presented as the mean ± SE of the mean, (n = 6).

Photodynamic treatment involves the uptake of photoactivatable molecules (photosensitizers) by cells and subsequent localized photo-irradiation with visible light at appropriate wavelength and dose. Optimization of both photoactivatable molecules concentrations and light doses with respect to the target cell response is required for an optimal photodynamic effect. The dynamic response of the MDA-MB-231 cancer cells, overexpressing NRP-1 receptor [31], exposed to increasing concentrations of NP-PS and various light doses was performed using the real-time impedance-based analysis **(** **Fig. 6** **)**. As expected, according to our findings from dark cytotoxicity analysis, no decrease in CI values was observed during the first 24 h post-exposure to NP-PS before light irradiation (**Fig. 6, left panel** **)**. However, CI kinetics obtained during the time interval of [25–55] h post-irradiation with various doses of light (1, 5 or 10 j/cm^2^) clearly showed discriminant profiles (**Fig. 6, right panel** **)**. According to nanoparticles concentrations and light doses, kinetic profiles showed a transient or a persistent decrease of NCI **(** **Fig. 6** **)**. Distinct phases of cell response along the post-irradiation period can be highlighted. For instance with low concentrations and/or light doses (*e.g.* 1 µM and 1 J/cm^2^), a transient decrease phase was characterized, followed by an increase until the end of the measurement. On the contrary, with high concentrations and/or light doses, immediate or late decrease of NCI can also be observed from the kinetics (**Fig. 6** **-B, right panel**), showing time-dependent heterogeneous profiles of cell response. Although metabolic tests and RTCA method assess distinct cell functions, comparable conclusions at a given time (*i.e.* 24 h post-irradiation) in term of cell survival were measured using a cell metabolism-based assay (WST-1) performed on the same plate (**Fig. 7**).

#### Model-based analysis of the photocytotoxic response

**Figure 8:**
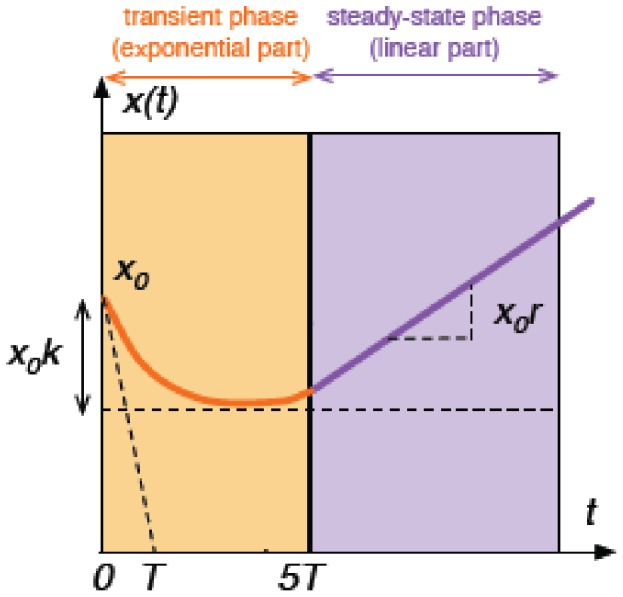
Exponential-Linear model structure of the transformed cell index (TCI) profile. The TCI is decomposed into two parts: a transient and a steady-state periods. This model is composed of three parameters (*T,k,r*) used as quantitative indicators in the therapeutic efficiency analysis. *T*, time constant of the transient phase; *K*, magnitude of the transient decrease; *r*, steady-state growth rate.

**Figure 9:**
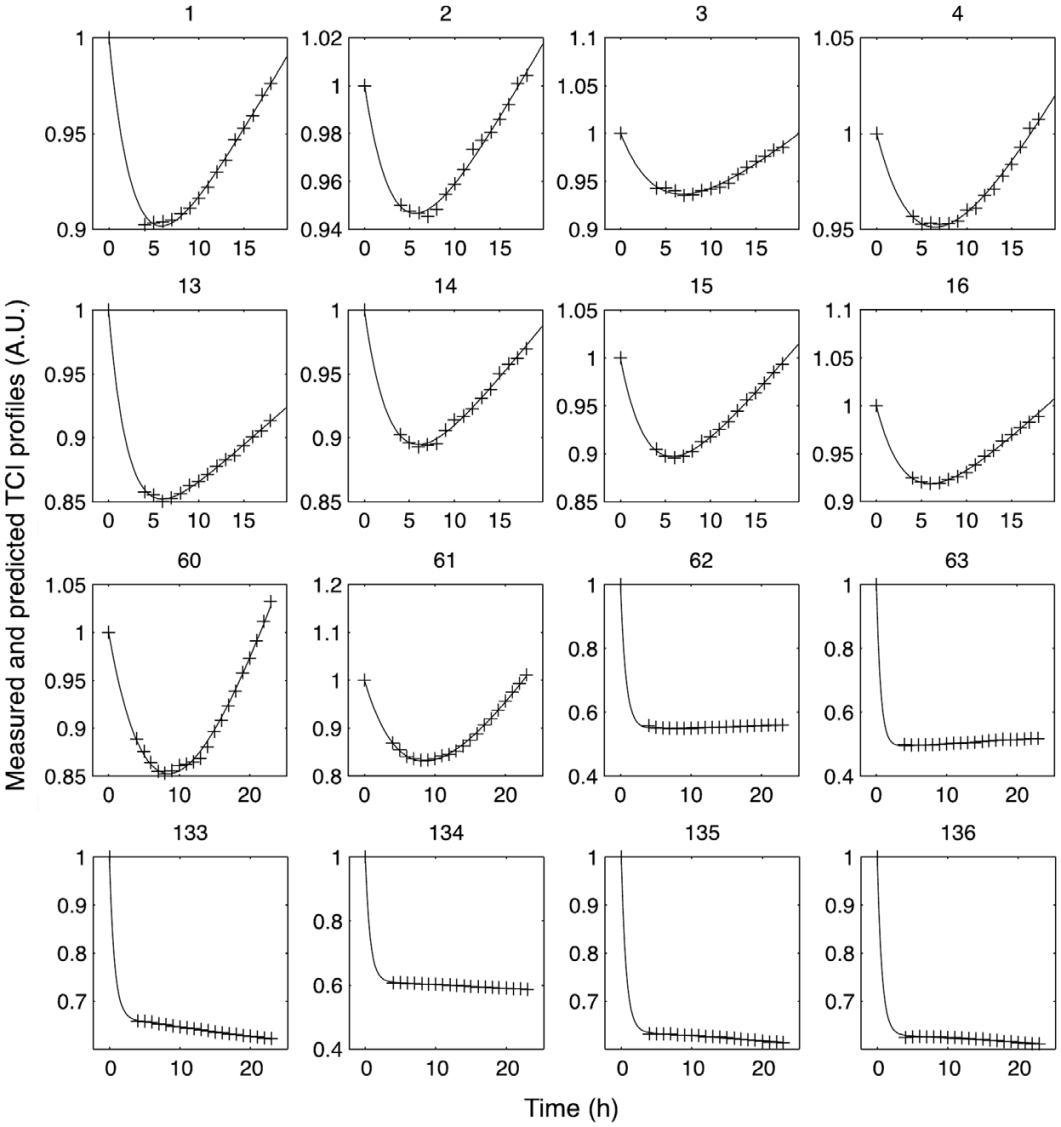
Model quality assessment. Comparison of measured (+) and predicted (−) responses for 16 cases among 144 time profiles of the transformed cell index (TCI). These 16 profiles sum up the variability of the cell response profiles observed between all the cell cultures.

To take a full advantage of all the information contained in the CI kinetics profiles of the post-irradiation period, we proposed to analyze the data by a parametric model, and to evaluate the model parameters as global quantitative characteristics of the complete *in vitro* cell response. The CI variable was firstly transformed into a new modeling variable called transformed cell index (TCI) to obtain quasi-linear steady-state behaviour of the CI profile. Such a variable transformation was already applied to *in vivo* data for tumor response modeling [32]. We showed that an exponential-linear equation can describe all the measured TCI kinetics profiles. This model structure is defined by three parameters; *r:* the steady-state growth rate; *T and K* as time constant and magnitude of the transient decrease of the TCI kinetics, respectively **(** **Fig. 8** **)**. These parameters appear as fingerprint characteristics of the photo-induced cell response; the exponential part corresponds to the transient decrease phase of the response while the linear part describes its steady-state trend. More importantly, a good consistency was obtained between the experimentally measured and the predicted response profiles, as illustrated by some examples presented in (**Fig. 9** **)**, corroborating the relevance of the proposed model.

**Figure 10:**
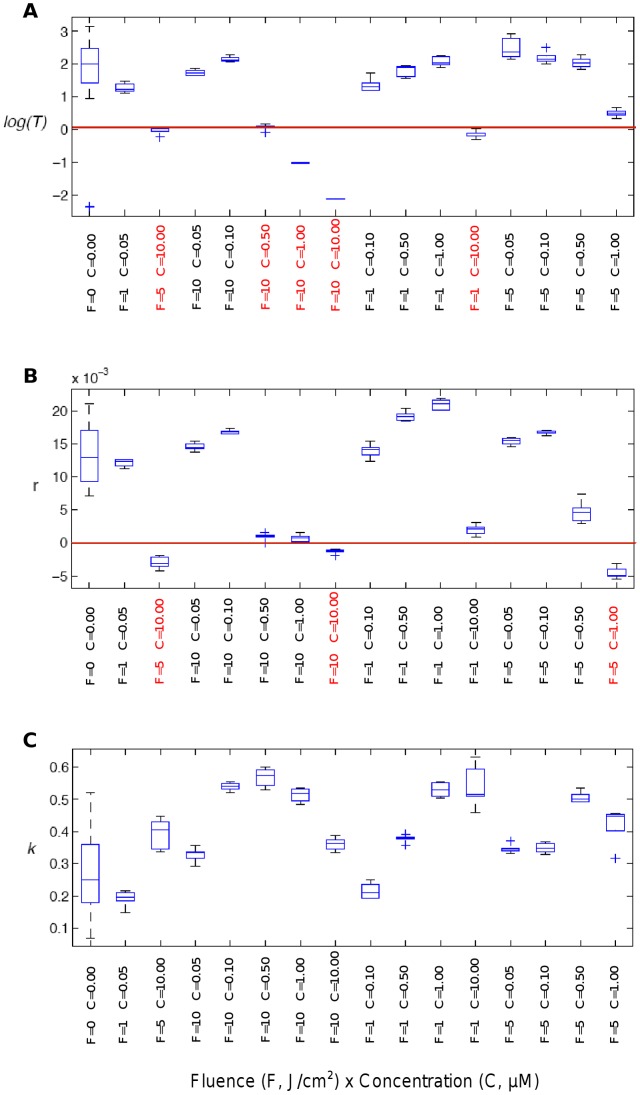
Synergic effects between the photosensitizer concentration, C, and light fluence, F, on the model parameters. The three model parameters (*T, r, k*) were analyzed with respect to C.F values. A) Any reduction of the time constant *T* suggests a faster decrease of the transient phase (positive therapeutic effect). Almost all the values of *T* below a reference threshold fixed to *log(T) = 0* correspond to a synergistic condition defined by C.F ≥5. B) Any reduction of the transformed cell index (TCI) growth rate *r* suggests a slow down of the steady-state growth rate during its post-transient phase (positive therapeutic effect). Conversely, any increase of it leads to locally degrading the therapeutic response. Almost all the values of *r* below a reference threshold fixed to *r = 0 (no steady-state growth)* correspond to a synergistic condition defined by C.F ≥5. C) Any positive enhancement of the magnitude of the transient decrease *k* suggests a deeper decrease of the TCI during its transient decrease (positive therapeutic effect). Conversely, any decrease of it leads to locally degrading the therapeutic response. The largest values of *k* all correspond to large values of the interaction between the concentration and the fluence.

The model parameters *T, K, r* were then used as numeric indicators of the *in vitro* photodynamic efficiency with respect to nanoparticles concentrations (C) and fluence levels (F) **(** **Fig. 10A–B** **–C, respectively)**. Low time constant values (*T*), defined as lower than a threshold given by *log T = 0,* were obtained for five photodynamic experimentations mentioned in red on the abscissa axis (**Fig. 10** **-A**), suggesting for these conditions a rapid decrease of the transient phase. These same conditions were also associated with low *r* values, defined as lower than *r* = 0 (**Fig. 10** **-B**), suggesting a slow down of the steady-state growth rate of the post-transient phase. The experimental conditions (C.F) corresponding to low *T* and *r* values also showed a tendency of correlation with the largest values of *K* (**Fig. 10** **-C**), indicating a deeper decrease of the TCI during the transient phase.

**Figure 11:**
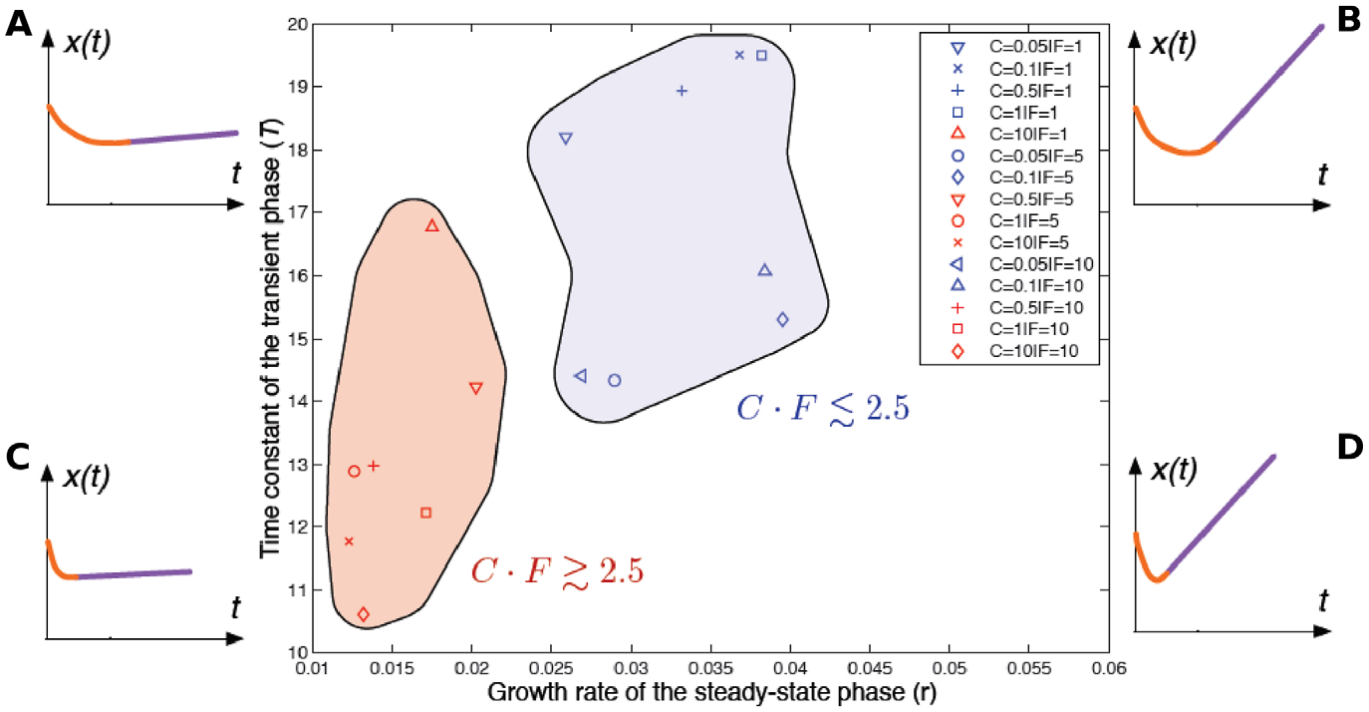
Profile of the photodynamic effects based on the time constant (*T*) and the growth rate (*r*). This synthetic representation describes both transient and steady-state effects. Corners correspond to four distinct scenarios of the therapeutic responses. The bottom left-hand corner (C) is the ideal case: a rapid decrease followed by a null steady-sate growth. All the estimates of *T* and *r* are projected in this map. Two distinct groups are described by red and blue regions. The most efficient group (red group) corresponds to the values of the concentration-fluence interaction *C.F* ≥2.5.

An efficient photodynamic effect was characterized by high *K*, low *T* and *r* values, suggesting a strong interaction between these three dynamic parameters and their capacity to characterize the *in vitro* photodynamic response. An ideal photodynamic response is characterized by a rapid and high decrease of the transient phase (low *T* or high *K*) followed by a null steady-state growth (*r* ≈ 0) **(** **Fig. 11** **,** insert C**)**. On the contrary, a lack of photodynamic efficiency is illustrated by a low decrease of the transient phase (high *T*) followed by an increase in the growth rate (high *r*) **(** **Fig. 11** **,** insert B**)**. Based on the different values of *T* and *r* parameters, two distinct groups of C.F synergistic effects were identified (**Fig. 11** **).** The most efficient experimental conditions **(** **Fig. 11** **,** red group**)** correspond to the interaction defined by C.F ≥2.5.

## Discussion

Using the impedance-based real-time analysis of cell proliferation with subsequent computational modeling of the experimentally obtained dynamic data, we characterized for the first time the *in vitro* photodynamic activity of multifunctional nanoparticles, used as photosensitizer delivery system. We optimized hybrid nanoparticles consisting of a gadolinium core, silica shell containing the covalently grafted chlorin photosensitizer, DOTAGA chelates as surfactant and ATWLPPR peptide as surface-localized targeting units.The dynamic analysis of cell response revealed that these optimized nanoplatforms containing photosensitizer induced discriminant profiles of photocytotoxicity kinetics in a photosensitizer concentration- and light dose-dependent manner. The nanoparticles conferred photosensitivity to cancer cells, providing evidence that the photosensitizer molecules grafted within the nanoparticle matrix can be photoactivated to yield photocytotoxic effects.

Although metabolic tests such as WST-1 assay and RTCA method measure distinct cell functions, comparable results were obtained using both tests, suggesting that the decrease in impedance values post-treatment was dominated by a decrease in cell viability characterizing the photodynamic efficiency of PDT. Positive relationships between both technologies have been previously described [21], [29], [30], suggesting the interest of combining both complementary approaches. The decrease in CI following PDT efficiency of nanoparticles may result from alterations of cell adhesion properties and impedance-based technologies such as the xCELLigence analyzer reflect cell parameters directly related to cells attachment [30]. Indeed, PDT can induce cell membrane damage and alteration in cancer cell adhesiveness mainly through the generated cytotoxic ROS (see review: [33]). Integrin-extracellular matrix (ECM) interactions lead to distinct cellular responses, such as cell proliferation, differentiation and migration. Runnels *et al.*, [34] investigated the effects of benzoporphyrin-derivative monoacid ring A-PDT on the cell adhesion properties of human ovarian cancer cell line. After photosensitization, the authors demonstrated a loss of integrins ability to bind to ECM proteins (*e.g.* collagen IV, fibronectin, laminin and vitronectin), both *in vitro* and *in vivo* concomitantly with a loss of β1 integrin-containing focal adhesion plaques. Benzoporphyrin-derivative monoacid ring A-PDT also interfered with the ability of fibroblasts to adhere to ECM components without altering integrin expression, but associated with the suppression of focal adhesion kinase phosphorylation [35]. Similar observations in cell migration and invasion abilities have been reported by Yang *et al.*, [36] in KJ-1 and Ca9-22 cancer cells after PDT. The expression of the intercellular adhesion molecule-1 (ICAM-1) and vascular cell adhesion molecule-1 (VCAM-1) were also down-regulated in endothelial cells after PDT [37]. The effect of PDT was also studied on cadherins, a class of adhesion molecules involved in the formation of stable junctions between cells in tissues. Under apoptotic conditions, zinc (II)-phthalocyanin-PDT induced a rapid disorganization of the E-cadherin-mediated cell-cell adhesion, with the detachment of cells from the substratum *via* β1 integrins [33].

The time effect on nanoparticles-mediated cellular impact is accepted as an important factor to consider when describing the response [38], [29]. Beside the concentration- and the light fluence-cellular effect, our study also demonstrated post-PDT time-dependent heterogeneous profiles of cell response, generating rich dynamic data. The parametric modeling analysis of the photocytotoxicity time profiles allowed the deduction of three dynamic model parameters (*r, T*, *k)* that completely characterize the dynamic cell response. The consistency between the experimentally measured data and the response profiles predicted by the model, testify to the predictive ability of the proposed model. The three model parameters describe distinct phases of the photodynamic cell response, and provide numeric information about the dynamic behaviour of cell response. Taken as numeric indicators, the estimated model parameters allowed evaluation of the photodynamic cell response according to the synergistic contribution/impact between the therapeutic factors (*i.e.* nanoparticles-grafted photosensitizer concentration and light fluence). More importantly, according to the synergistic effects between photosensitizer concentration and light fluence, a relationship was observed between the three dynamic model parameters, indicating their potential to characterize the photodynamic cell response.

*In vitro* photodynamic activity of photoactivatable molecules is usually evaluated by the determination of the median lethal light dose (DL_50_) at a given time-point and for each concentration of photosensizer, which is often quite approximate and hard to perform. Thereby, our present study using more than one quantitative parameter and more accurate quantification of the global photodynamic response provides powerful results. Although there is a growing interest for the RTCA using impedance technology, to our knowledge only two articles [39], [40] have recently tried to describe the dynamic cell behaviours using mathematical and computational modeling of the impedance-derived dynamic data. Chen et *al.*, [40] suggested an exponential time function model, reflecting profile of fibroblastic cell proliferation without any treatment. Different model parameters were estimated by fitting the measured impedance dynamic data to the proposed model. But, the proposed exponential model is not suited to describe treatment responses. Eisenberg et *al.*, [39] characterized the precise role of myoferlin protein in cancer cell invasion using a combination of mathematical modeling and real-time impedance-based cell invasion assay. The proposed mathematical model is used to confirm or invalidate some hypothesis, owing to model simulations but little information is available about the parameters estimation method.

In the absence of light irradiation after nanoparticles exposure, we observed alterations in cell proliferation even for low concentrations of nanoparticles. We can annotate that MTT test only showed a cytotoxic effect with the highest concentration of 10 µM. This discrepancy already reported in the literature, may be mainly due to the fact that both assays measure distinct cellular activities. MTT test detects cell viability through mitochondrial metabolism, while RTCA considers cell death or proliferation, cell size, cell morphology, and cell adhesion to the well bottom as defined by cells-microelectrode contact surface [41], [30], [42], [26], [43]. This observation raises the question about the underlying mechanism of nanoparticles interaction on cellular activities. Numerous research groups have pointed out the difficulty of using the classical single-point techniques usually based on cell metabolism activity to assess nanoparticules-related cytotoxicity [25], [38]. For instance, the nanoparticles as well as nanoparticles-generated ROS can indeed interact with mitochondrial enzymes activity and disturb mitochondrial metabolism-based assays. Lactate dehydrogenase cell viability assay can be affected by nanoparticles which can bind to the enzyme and impede its release into the extracellular medium [26], [23], [44]. Moreover, the time-dependent nanocytotoxic effects have been previously reported [38], [29]. The impedance-based real-time cell analysis also revealed a late alteration of cell proliferation/adhesion induced by the nanoparticles in darkness. Such delayed cellular effect may be explained by the time-dependent cellular uptake process with such nanoparticles [45], and that nanocytotoxicity may be related to this uptake [25], [29]. Thereby, as label-free method the impedance-based real-time cell analysis method offers advantages over the cell metabolism-based end-point assays, allowing continuous monitoring of nanocytotoxicity and discrimination between early and late cellular effects.

Overall, this study validates a novel approach for continuous monitoring and accurate quantification of the dynamics of cell response using nanoparticles. According to our results and as suggested by others, metabolic tests may not be adapted to measure biological effect induced by low concentrations of nanoparticles [25] compared to the impedance-based real-time cell analysis system. The computational analysis appears as a useful tool for better understanding the dynamics of cell behaviour, such as nanoparticles-cells interactions. This analysis approach provides insights for rapid and accurate evaluation of *in vitro* dynamic cell response, which may help to adequately address investigations about the nanoparticle-mediated cellular effects. For the first time, the model-based approach we proposed in this study, successfully characterize the treatment response at every time point. Additionally, it provides a simple statistical analysis, focusing on only three characteristic parameters.

## Methods

### Experimental Procedure: Nanoparticles Synthesis

The approach developed here is based on multifunctional silica-based nanoparticles, used as photosensitizer carriers and grafted to the peptide ATWLPPR targeting vascular receptor NRP-1 [31], [14]. Multifunctional silica-based nanoparticles were thus designed and consisted of a surface grafted tumor targeting peptide H-Ala-Thr-Trp-Leu-Pro-Pro-Arg-OH (called ATWLPPR) and encapsulated a photosensitizer (PS: a chlorin, (5-(4-carboxyphenyl)-10,15,20-triphenyl-chlorin, TPC) and a MRI contrast agent (gadolinium DOTAGA chelates). These nanoparticles were noted NP-PS. To assess the nanoparticles for their own cellular effects, nanoparticles without photosensitizer and without surface targeting peptide were also synthesized and considered as control noted NP. The synthesis of gadolinium nanoparticles embedded in a polysiloxane shell has been previously and widely described by our group [13], [46]–[49]. The top down process leading from the gadolinium oxide particles to the ultrasmall polysiloxane particles with gadolinium chelates at the surface has been recently published [49], [50]. Briefly, we optimized ATWLPPR-targeted silica-based small nanoparticles grafted by gadolinium chelates for MRI and a chlorin as a photosensitizer. The nanoparticles were synthesized by a top down process. The first step is the formation of a gadolinium oxide core by a modified polyol route in diethylene glycol followed by the growth of the polysiloxane shell by a sol-gel process and then by the grafting of DOTAGA (1,4,7,10-tetraazacyclododecane-1-glutaric anhydride-4,7,10-triacetic) acid to the inorganic matrix *via* an amide function. The photosensitizer is added to the nanoparticles during the sol gel process by direct coupling of the photosensitizer to a silane precursor. The dissolution of the gadolinium core and the entrapping of the gadolinium by the chelates occur during the purification in water leading to ultrasmall nanoparticles with a size inferior to 5 nanometers [50]. The ATWLPPR targeting peptide is finally grafted on the DOTAGA *via* an amide function [49].

### Experimental Procedure: Cell Line Culture

To investigate the potential of silica-based nanoparticles to induce *in vitro* photodynamic effect on tumor cells, MDA-MB-231 human breast cancer cells over-expressing the vascular NRP-1 receptor were used. MDA-MB-231 cells were purchased from American Type Culture Collection (ATCC, Manassas, VA, USA). Cells were routinely grown in Roswell Park Memorial Institute (RPMI 1664) medium (Invitrogen, France) supplemented with 9% heat-inactivated fetal bovine serum (PAN™ Biotech GmbH, Germany), 100 U/mL penicillin and 100 µg/mL streptomycin (Invitrogen, France), and 2 mM L-Glutamine (Invitrogen, France) in a controlled atmosphere of 5% CO_2_, 95% humidified air at 37°C in 75 cm^2^ culture flasks. Cell culture materials were purchased from Costar (Dutscher, Brumath, France).

### Experimental Procedure: Cytotoxicity and Photocytotoxicity Studies

*In vitro* cytotoxicity and photo-induced cytotoxic effects of the synthesized nanoparticles were investigated on the MDA-MB-231 cancer cells by using the standard cell metabolism-based cell viability assays (MTT and WST-1) and the real-time impedance-based analysis.

#### Real-time impedance-based cell analysis

MDA-MB-231 cells attachment, proliferation and size variations were monitored in real-time and measured as impedance using 96-well E-Plates™ and the xCELLigence system (Real Time Cell Analyzer Single Plate (RTCA SP®) system). The xCELLigence system was developed by ACEA Biosciences in conjunction with Roche Diagnostics GmbH (Roche Applied Science, Mannheim, Germany). The technology uses microwell plates whose bottoms are covered with microelectrodes as an electrical impedance cell sensor to measure the level of impedance on the surface of cell culture plate/well, which corresponds to the extend of cell-covered area. This method is label-free and allows real-time, automatically and continually monitoring of cellular status changes (*e.g.* adhesion, proliferation, morphology, viability) during the whole process of cell-reagent interaction. It is based on measurement of electrical impedance created by attached cells across the high-density electrode array coating the bottom of the wells [41]. Impedance value is automatically converted to a dimentionless parameter CI that is defined as relative change in electrical impedance created by cells. CI value represents cellular status and is directly proportional to number, proliferation, size, morphology, and attachment forces of the cells. As cells detach and die (i.e. during cytotoxic events) CI values decrease. The RTCA SP Station was connected to the RTCA Analyzer and subsequently joined the RTCA Control Unit. The xCELLigence system was connected and the RTCA SP Station, in which the culture E-Plate is mounted, was placed inside the incubator at 37°C and 5% CO_2_. Then, a self check for proper electrical contact using RTCA Resistor Plate 96 was conducted prior to any experiment. All measurements were controlled by the RTCA software 1.2.1 (Roche Diagnostics).

For measurements, background impedance of the E-Plate was first determined before seeding the cells by the addition of 50 µL culture medium to each well and subtracted automatically by the RTCA software following the equation: CI = (Z_i_-Z_0_)/15 with Z_i_ as the impedance at any given time point and Z_0_ as the background signal [51]. Subsequently, a 150 µL cell suspension containing 10^4^ MDA-MB-231 cells was seeded in each well, and allowed to settle at the bottom of wells for 20 min before starting impedance measurement in 15 min intervals. 24 h after seeding, 10 µL of growth medium with or without increasing final concentrations of photosensitizer (0.05, 0.10, 0.50, 1.00 or 10.00 µM) in NP-PS or the corresponding final concentrations of gadolinium (2.9, 5.8, 29.2, 58.5 or 585.0 µM) for the control NP was added in each well, and the cultures were kept in darkness. Each concentration was tested in sixplicate. To allow nanoparticles internalization, the cells were grown for further 24 h during which impedance was measured every 15 min. Then, the cells were washed three times with growth medium to remove the un-internalized nanoparticles. The medium was renewed by adding 150 µL of fresh growth medium. After that, the cells were either exposed to various doses of light (1, 5, or 10 J/cm^2^) using a diode laser, Ceralas PDT 652 (CeramOptec GmbH, Biolitec, Germany) to assess the photo-induced cyto-toxicity of the NP-PS, or let without irradiation for the determination of dark cytotoxicity of the nanoparticles. Irradiation was carried out at 652 nm with an irradiance of 4.54 mW/cm^2^. The cell growth was then monitored for the indicated time, and impedance was measured every 15 min. Based on impedance measurements, CI values were automatically derived and recorded as a function of time from the time of plating until the end of the experiments.

#### MTT and WST-1 cell metabolism-based assays

Cell proliferation and survival after incubation with the various concentrations of nanoparticles was also measured using standard single end-point metabolic assays; 3-(4,5-dimethylthiazol-2-yl)-2,5-diphenyl tetrazolium bromide (MTT) and 4-[3-(4-lodophenyl)-2-(4-nitrophenyl)-2H-5-tetrazolio]-1,3-benzene disulfonate (WST-1). These colorimetric assays determine the mitochondrial metabolic activity by measuring the reduction of the tetrazolium salt MTT or WST-1 to formazan cristals by mitochondrial dehydrogenases in viable cells.

To compare the photo-induced cellular effect measured with the impedance-based technology at the endpoint of the assay (∼25 h post-irradiation), WST-1 test was optimized and performed at the same time point on the same E-Plate. Briefly, at the end of the impedance measurement (∼25 h post-irradiation), 15 µL of cell proliferation reagent WST-1 (Roche) was added in each well of the E-Plate (1∶10 final dilution, according to the manufacturer’s instructions). The cells were then incubated at 37°C for 1 h. Formazan cristal generated from the reduction of WST-1 salt was quantified by measuring the absorbance at 450 nm against a background control consisting of 150 µL medium and 15 µL cell proliferation reagent WST-1, using a Multiskan microplate reader (Labsystem, Cergy-Pontoise, France).

In parallel, MTT single-point test was also performed to assess the dark cytotoxicity of the nanoparticles using standard 96-well flat-bottomed microtiter plates, as previously described [49]. Similarly to WST-1 assay, cell viability was expressed as the percentage of the controls cultivated under the same conditions without nanoparticles exposure.

### Statistical Methods/Model-based Analysis of the Dynamic Data

#### Design of experiments

As described above in the experimental procedure, we investigated the cytotoxicity of the NP-PS and NP, and the photocytotoxic activity of the nanoparticles-containing photosensitizer (NP-PS) in dynamic manner using the impedance-based technology. Two therapeutic factors of PDT were examined in this study:

- the fluence (spatial density of light energy, or dose of light), noted *F*, decomposed into four levels: {0; 1; 5; 10} J/cm^2^;
- and the concentration of the photosensitizing agent grafted in the nanoparticles, noted *C*, decomposed into six levels: {0.00, 0.05, 0.10, 0.5, 1.00, 10.00} µM, or the corresponding concentrations of gadolinium {0.0; 2.9; 5.8; 29.2; 58.5; 585.0} µM in the control NP.

A full factorial design of experiments, composed of 24 different treatments repeated six times each (six wells per treatment), was performed to estimate and compare the effects caused by these two factors and their potential synergy on the PDT dynamic response. This response was measured by the cell index provided by the impedance-based real-time cell analysis system (xCELLigence®, Roche diagnostics). This generation of biochips may rapidly generate a large amount of time profiles and a first challenge is to provide statistical techniques able to reduce the dimension of the inference problem. Two approaches have been developed herein for two different applications: the automatic selection of time regions of interest for cyto-toxicity analysis, and a parametric modeling of the CI kinetics profiles for the photocyto-toxic response analysis.

#### Selection of time regions of interest

In a first step, a study area is selected: [25–120] h for a statistical analysis among the whole measurement range, Fig. 3. Secondly, all the CI profiles are normalized at one at t_0_ = 25 h. A multivariate analysis of variance (MANOVA) associated with a canonical analysis is then applied to the normalized CI values in order to identify the time regions of interest for which the largest separation between the six groups of concentration may be observed, *i.e.* the most informative parts. Results are presented in Fig. 4. All the measurement time instants are projected into a canonical map, which summarizes more than 95% of the total variance information contained in the original data. The first canonical axis reveals the importance of a first time region of interest (blue group G1) corresponding to the end of the experimentation period (T ≈ 120 h). According to the second axis, another group (G2) has to be selected around T ≈ 45 h. These two time regions have been identified for the two sets of experimentation (nanoparticle with or without photosensitizer). In a fourth step, two one-way ANOVA are carried out upon the NCI values at time instants T1 = 45 h and T2 = 120 h for the six groups of concentration. Results are described by boxplots diagrams in Fig. 5.

#### Model-based analysis of the photocytotoxic response

The CI variable *y(t)* corresponding to the post-irradiation period is firstly transformed into a new modeling variable *x(t)*, entitled transformed cell index (TCI) and defined as.
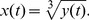
(1)


This transformation is similar to the one previously proposed [32] to obtain a quasi-linear steady-state behaviour of the CI profile. To benefit from all the information contained in the TCI profiles, we propose to analyze them by a parametric model and to analyze the parameters as global characteristics of the complete *in vitro* response. In this application, the proposed model structure relies on an exponential-linear equation:
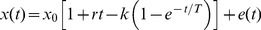
(2)where *x_0_* is the initial value of the response; *r* is the steady-state growth rate; *T, k* are the time constant and the magnitude of the transient decrease respectively and *e*(*t*) is the random output error (experimental variability) defined by a Gaussian distribution with a null mean and a standard deviation *σ*. These three model parameters completely describe the kinetics profile plotted in Fig. 8. The exponential part: *x*_0_ *k*(1−*e^−t/T^*) corresponds to the transient phase of the response while the linear part: *x*(*t*) =  *x*_0_ *rt* describes the steady-state trend of the TCI kinetics. The quality of predicted response is emphasized in Figure 9. *r, k* and *T* are used thereafter as numeric indicators of the *in vitro* PDT response.

#### Parameter estimation

The parameter estimation of the model parameters *(r,k,T)* is carried out by nonlinear optimization algorithms implemented into the computational environment Matlab^©^.

## References

[1] Praetorius NP, Mandal TK (2007) Engineered nanoparticles in cancer therapy. Recent Pat Drug Deliv Formul 1: 37–51. 19075873 10.2174/187221107779814104

[2] Hong H, Zhang Y, Sun J, Cai W (2009) Molecular imaging and therapy of cancer with radiolabeled nanoparticles. Nano Today 4: 399–413. 20161038 10.1016/j.nantod.2009.07.001 PMC2753977

[3] Koo H, Huh MS, Sun IC, Yuk SH, Choi K, et al (2011) In vivo targeted delivery of nanoparticles for theranosis. Acc Chem Res 44: 1018–1028. 21851104 10.1021/ar2000138

[4] Hong S, Leroueil PR, Janus EK, Peters JL, Kober MM, et al (2006) Interaction of polycationic polymers with supported lipid bilayers and cells: nanoscale hole formation and enhanced membrane permeability. Bioconjug Chem 17: 728–734. 16704211 10.1021/bc060077y

[5] Caruso G, Caffo M, Alafaci C, Raudino G, Cafarella D, et al (2011) Could nanoparticle systems have a role in the treatment of cerebral gliomas? Nanomedicine 7: 744–752. 21419873 10.1016/j.nano.2011.02.008

[6] Mohs AM, Provenzale JM (2010) Applications of nanotechnology to imaging and therapy of brain tumors. Neuroimaging Clin N Am 20: 283–292. 20708547 10.1016/j.nic.2010.04.002

[7] Solomon M, D'Souza GG (2011) Recent progress in the therapeutic applications of nanotechnology. Curr Opin Pediatr 23: 215–220. 21412081 10.1097/MOP.0b013e32834456a5

[8] Bechet D, Couleaud P, Frochot C, Viriot ML, Guillemin F, et al (2008) Nanoparticles as vehicles for delivery of photodynamic therapy agents. Trends Biotechnol 26: 612–621. 18804298 10.1016/j.tibtech.2008.07.007

[9] Sun C, Lee JS, Zhang M (2008) Magnetic nanoparticles in MR imaging and drug delivery. Adv Drug Deliv Rev 60: 1252–1265. 18558452 10.1016/j.addr.2008.03.018 PMC2702670

[10] McCarthy JR, Weissleder R (2008) Multifunctional magnetic nanoparticles for targeted imaging and therapy. Adv Drug Deliv Rev 60: 1241–1251. 18508157 10.1016/j.addr.2008.03.014 PMC2583936

[11] Tassa C, Shaw SY, Weissleder R (2011) Dextran-coated iron oxide nanoparticles: a versatile platform for targeted molecular imaging, molecular diagnostics, and therapy. Acc Chem Res 44: 842–852. 21661727 10.1021/ar200084x PMC3182289

[12] Chouikrat R, Seve A, Vanderesse R, Benachour H, Barberi-Heyob M, et al (2012) Non polymeric nanoparticles for photodynamic therapy applications: recent developments. Curr Med Chem 19: 781–792. 22214454 10.2174/092986712799034897

[13] Faure AC, Dufort S, Josserand V, Perriat P, Coll JL, et al (2009) Control of the in vivo biodistribution of hybrid nanoparticles with different poly(ethylene glycol) coatings. Small 5: 2565–2575. 19768700 10.1002/smll.200900563

[14] Couleaud P, Bechet D, Vanderesse R, Barberi-Heyob M, Faure AC, et al (2011) Functionalized silica-based nanoparticles for photodynamic therapy. Nanomedicine (Lond) 6: 995–1009. 21726134 10.2217/nnm.11.31

[15] Weishaupt KR, Gomer CJ, Dougherty TJ (1976) Identification of singlet oxygen as the cytotoxic agent in photoinactivation of a murine tumor. Cancer Res 36: 2326–2329. 1277137

[16] Fingar VH (1996) Vascular effects of photodynamic therapy. J Clin Laser Med Surg 14: 323–328. 9612199 10.1089/clm.1996.14.323

[17] Dougherty TJ, Gomer CJ, Henderson BW, Jori G, Kessel D, et al (1998) Photodynamic therapy. J Natl Cancer Inst 90: 889–905. 9637138 10.1093/jnci/90.12.889 PMC4592754

[18] Verma S, Watt GM, Mai Z, Hasan T (2007) Strategies for enhanced photodynamic therapy effects. Photochem Photobiol 83: 996–1005. 17880492 10.1111/j.1751-1097.2007.00166.x

[19] Chatterjee DK, Fong LS, Zhang Y (2008) Nanoparticles in photodynamic therapy: an emerging paradigm. Adv Drug Deliv Rev 60: 1627–1637. 18930086 10.1016/j.addr.2008.08.003

[20] Brevet D, Gary-Bobo M, Raehm L, Richeter S, Hocine O, et al.. (2009) Mannose-targeted mesoporous silica nanoparticles for photodynamic therapy. Chem Commun (Camb) 1475–1477. 10.1039/b900427k 19277361

[21] Vistejnova L, Dvorakova J, Hasova M, Muthny T, Velebny V, et al (2009) The comparison of impedance-based method of cell proliferation monitoring with commonly used metabolic-based techniques. Neuro Endocrinol Lett 1: 121–127. 20027157

[22] McGowan EM, Alling N, Jackson EA, Yagoub D, Haass NK, et al (2011) Evaluation of cell cycle arrest in estrogen responsive MCF-7 breast cancer cells: pitfalls of the MTS assay. PLoS One 6: e20623. 21673993 10.1371/journal.pone.0020623 PMC3108819

[23] Elisia I, Popovich DG, Hu C, Kitts DD (2008) Evaluation of viability assays for anthocyanins in cultured cells. Phytochem Anal 19: 479–486. 18435529 10.1002/pca.1069

[24] Barbu E, Molnàr E, Tsibouklis J, Górecki DC (2009) The potential for nanoparticle-based drug delivery to the brain: overcoming the blood-brain barrier. Expert Opin Drug Deliv 6: 553–565. 19435406 10.1517/17425240902939143

[25] Soenen SJ, Rivera-Gil P, Montenegro JM, Parak WJ, De Smedt SC, et al (2011) Cellular toxicity of inorganic nanoparticles: Common aspects and guidelines for improved nanotoxicity evaluation. Nano Today 6: 446–465.

[26] Soenen SJ, De Cuyper M (2009) Assessing cytotoxicity of (iron oxide-based) nanoparticles: an overview of different methods exemplified with cationic magnetoliposomes. Contrast Media Mol Imaging 4: 207–219. 19810053 10.1002/cmmi.282

[27] Gupta S, Dwarakanath BS, Muralidhar K, Koru-Sengul T, Jain V (2010) Non-monotonic changes in clonogenic cell survival induced by disulphonated aluminium phthalocyanine photodynamic treatment in a human glioma cell line. J Transl Med 8: 43. 20433757 10.1186/1479-5876-8-43 PMC2885318

[28] Winther J (1989) Photodynamic therapy effect in an intraocular retinoblastoma-like tumour assessed by an in vivo to in vitro colony forming assay. Br J Cancer 59: 869–872. 2525401 10.1038/bjc.1989.184 PMC2246719

[29] Li J, Guo D, Wang X, Wang H, Jiang H, et al (2010) The Photodynamic Effect of Different Size ZnO Nanoparticles on Cancer Cell Proliferation In Vitro. Nanoscale Res Lett 5: 1063–1071. 20671778 10.1007/s11671-010-9603-4 PMC2893699

[30] Quereda JJ, Martínez-Alarcón L, Mendoça L, Majado MJ, Herrero-Medrano JM, et al (2010) Validation of xCELLigence real-time cell analyzer to assess compatibility in xenotransplantation with pig-to-baboon model. Transplant Proc 42: 3239–3243. 20970663 10.1016/j.transproceed.2010.05.059

[31] Thomas N, Pernot M, Vanderesse R, Becuwe P, Kamarulzaman E, et al (2010) Photodynamic therapy targeting neuropilin-1: Interest of pseudopeptides with improved stability properties. Biochem Pharmacol 80: 226–235. 20380812 10.1016/j.bcp.2010.03.036

[32] Bastogne T, Samson A, Vallois P, Wantz-Mézières S, Pinel S, et al (2010) Phenomenological modeling of tumor diameter growth based on a mixed effects model. J Theor Biol 262: 544–552. 19835891 10.1016/j.jtbi.2009.10.008

[33] Pazos MdC, Nader HB (2007) Effect of photodynamic therapy on the extracellular matrix and associated components. Braz J Med Biol Res 40: 1025–1035. 17665038 10.1590/s0100-879x2006005000142

[34] Runnels JM, Chen N, Ortel B, Kato D, Hasan T (1999) BPD-MA-mediated photosensitization in vitro and in vivo: cellular adhesion and beta1 integrin expression in ovarian cancer cells. Br J Cancer 80: 946–953. 10362101 10.1038/sj.bjc.6690448 PMC2363035

[35] Margaron P, Sorrenti R, Levy JG (1997) Photodynamic therapy inhibits cell adhesion without altering integrin expression. Biochim Biophys Acta 1359: 200–210. 9434126 10.1016/s0167-4889(97)00115-8

[36] Yang TH, Chen CT, Wang CP, Lou PJ (2007) Photodynamic therapy suppresses the migration and invasion of head and neck cancer cells in vitro. Oral Oncol 43: 358–365. 16920382 10.1016/j.oraloncology.2006.04.007

[37] Volanti C, Gloire G, Vanderplasschen A, Jacobs N, Habraken Y, et al (2004) Downregulation of ICAM-1 and VCAM-1 expression in endothelial cells treated by photodynamic therapy. Oncogene 23: 8649–8658. 15467759 10.1038/sj.onc.1207871

[38] Mariani V, Ponti J, Giudetti G, Broggi F, Marmorato P, et al (2012) Online monitoring of cell metabolism to assess the toxicity of nanoparticles: The case of cobalt ferrite. Nanotoxicology 6: 272–287. 21495878 10.3109/17435390.2011.572302

[39] Eisenberg MC, Kim Y, Li R, Ackerman WE, Kniss DA, et al (2011) Mechanistic modeling of the effects of myoferlin on tumor cell invasion. Proc Natl Acad Sci USA 108: 20078–20083. 22135466 10.1073/pnas.1116327108 PMC3250187

[40] Chen SW, Yang JM, Yang JH, Yang SJ, Wang JS (2012) A computational modeling and analysis in cell biological dynamics using electric cell-substrate impedance sensing (ECIS). Biosens Bioelectron 33: 196–203. 22261483 10.1016/j.bios.2011.12.052

[41] Kirstein SL, Atienza JM, Xi B, Zhu J, Yu N, et al (2006) Live cell quality control and utility of real-time cell electronic sensing for assay development. Assay Drug Dev Technol 4: 545–553. 17115925 10.1089/adt.2006.4.545

[42] Heng BC, Das GK, Zhao X, Ma LL, Tan TT, et al (2010) Comparative cytotoxicity evaluation of lanthanide nanomaterials on mouse and human cell lines with metabolic and DNA-quantification assays. Biointerphases 5: FA88–97. 21171718 10.1116/1.3494617

[43] Hanusová V, Králová V, Schröterová L, Trilecová L, Pakostová A, et al (2010) The effectiveness of oracin in enhancing the cytotoxicity of doxorubicin through the inhibition of doxorubicin deactivation in breast cancer MCF7 cells. Xenobiotica 40: 681–690. 20698750 10.3109/00498254.2010.508821

[44] Soenen SJ, Brisson AR, De Cuyper M (2009) Addressing the problem of cationic lipid-mediated toxicity: the magnetoliposome model. Biomaterials 30: 3691–3701. 19371948 10.1016/j.biomaterials.2009.03.040

[45] Liu Y, Chen Z, Liu C, Yu D, Lu Z, et al (2011) Gadolinium-loaded polymeric nanoparticles modified with Anti-VEGF as multifunctional MRI contrast agents for the diagnosis of liver cancer. Biomaterials 32: 5167–5176. 21521627 10.1016/j.biomaterials.2011.03.077

[46] Bazzi R, Flores-Gonzalez MA, Louis C, Lebbou K, Dujardin C, et al (2003) Synthesis and luminescent properties of sub-5-nm lanthanide oxides nanoparticles. Journal of Luminescence 102: 445–450.

[47] Bazzi R, Flores MA, Louis C, Lebbou K, Zhang W, et al (2004) Synthesis and properties of europium-based phosphors on the nanometer scale: Eu2O3, Gd2O3:Eu, and Y2O3:Eu. J Colloid Interface Sci 273: 191–197. 15051451 10.1016/j.jcis.2003.10.031

[48] Louis C, Bazzi R, Marquette CA, Bridot JL, Roux S, et al (2005) Nanosized hybrid particles with double luminescence for biological labelling. Chem Mater 17: 1673–1682.

[49] Benachour H, Sève A, Bastogne T, Frochot C, Vanderesse R, et al.. (2012) Multifunctional peptide-conjugated hybrid silica nanoparticles for photodynamic therapy and MRI. Theranostics. In press. 10.7150/thno.4754 PMC3475218 23082101

[50] Lux F, Mignot A, Mowat P, Louis C, Dufort S, et al (2011) Ultrasmall rigid particles as multimodal probes for medical applications. Angew Chem Int Ed Engl 50: 12299–12303. 22057640 10.1002/anie.201104104

[51] Urcan E, Haertel U, Styllou M, Hickel R, Scherthan H, et al (2010) Real-time xCELLigence impedance analysis of the cytotoxicity of dental composite components on human gingival fibroblasts. Dent Mater 26: 51–58. 19767088 10.1016/j.dental.2009.08.007

